# Interfacial coupling-modulated ultraviolet photoresponse in WS_2_/R6G hybrid structure with synapse-like characteristics

**DOI:** 10.1016/j.isci.2026.116621

**Published:** 2026-06-30

**Authors:** Wenyu Xu, Lin Jiang, Qinyong He, Daliao Tao, Tengfei Yan

**Affiliations:** 1 Shanghai Engineering Research Center for Integrated Circuits and Advanced Display Materials, School of Microelectronics, Shanghai University, Shanghai 201800, China; 2 Polariton (Shanghai) Optoelectronic Technology Co., Ltd., Huancheng Road, Shanghai, China

**Keywords:** WS2, photodetector, synaptic behavior

## Abstract

Hybrid structures integrating two-dimensional semiconductors with organic molecules offer an effective platform for tuning interfacial carrier dynamics and optoelectronic functionality. Here, we report a WS_2_/Rhodamine 6G (R6G) hybrid photodevice based on mechanically exfoliated monolayer WS_2_ modified with R6G molecules. Under 375 nm illumination, the hybrid device exhibits a significantly enhanced ultraviolet photoresponse relative to pristine WS_2_, with a responsivity of up to 2.1 A/W and a detectivity approaching 1.0 × 10^12^ Jones. The device also shows clear synapse-like photoresponse characteristics, including excitatory postsynaptic current, pulse-number-dependent response, and frequency-dependent plasticity. Spectroscopic studies indicate that R6G modification substantially alters the exciton and carrier relaxation pathways of WS_2_. Time-resolved measurements reveal that R6G modification alters the exciton relaxation process in WS_2_, indicating interfacial carrier transfer from WS_2_ to R6G. This interfacial process contributes to the enhanced photodetection performance and enables the synapse-like photoresponse of the hybrid device.

## Highlights

• Interfacial coupling enhances ultraviolet photoresponse in WS_2_/R6G
• R6G modification enables light-driven synaptic plasticity
• Ultrafast spectroscopy reveals modified exciton relaxation pathways
• Interfacial charge transfer regulates carrier dynamics in WS_2_

## Graphical abstract

**Figure:**
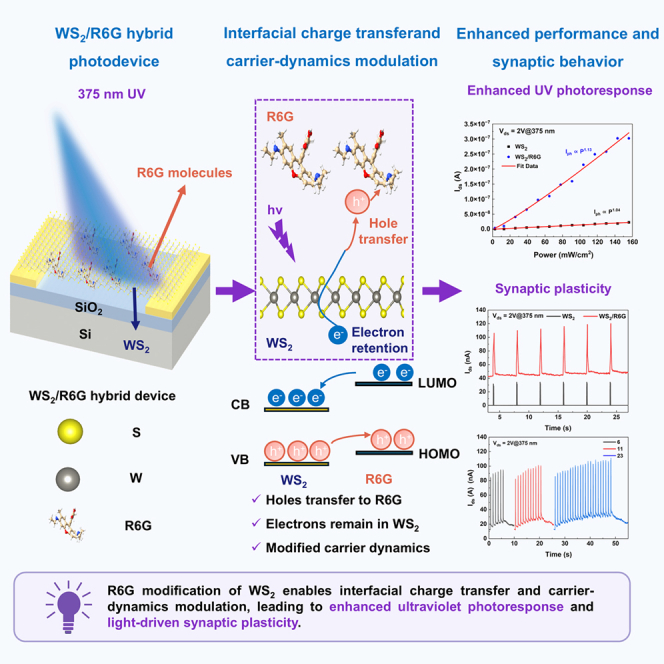

## Teaser

Optoelectronics; Condensed matter physics; Nanotechnology

## Introduction

Conventional computing architectures based on the von Neumann paradigm suffer from the physical separation of data storage and information processing, which results in high energy consumption and limited computational efficiency in data-intensive tasks.^1^^,^^2^ Neuromorphic electronics, inspired by biological neural networks, provide an attractive route to overcome this bottleneck by integrating signal processing and memory functions within a single device platform.^3^^,^^4^^,^^5^ In particular, optoelectronic synapses use light as the external stimulus to mimic synaptic plasticity and have attracted increasing interest because they integrate sensing, memory, and computation. Under pulsed illumination, these devices can exhibit photocurrent enhancement, decay, and accumulation, with their conductance modulated by the number and frequency of optical pulses, thereby resembling biological synaptic plasticity.^6^^,^^7^^,^^8^ In addition to pulse-number- and frequency-dependent current accumulation, paired-pulse facilitation (PPF) is a widely used and important parameter for evaluating short-term plasticity in artificial synaptic devices, because it directly reflects the enhancement of the postsynaptic current induced by two consecutive stimuli. Recent optoelectronic synapses, including MoS_2_/ZnO heterostructure-based devices, vacancy-engineered ZnO nanocage photoelectrochemical synapses, and diamond-based neuromorphic retina perception systems, have employed PPF measurements to verify pulse-interval-dependent current facilitation and short-term synaptic plasticity.^9^^,^^10^^,^^11^

Among the candidate materials for optoelectronic synapses, two-dimensional transition metal dichalcogenides (TMDs) are especially appealing owing to their atomic thickness, strong light-matter interaction, and tunable electronic structures.^12^^,^^13^^,^^14^^,^^15^ Monolayer WS_2_ is a representative direct-bandgap semiconductor that exhibits strong excitonic effects and pronounced optical absorption in the visible region, making it suitable for photodetection and light-modulated electronic applications.^16^^,^^17^^,^^18^ However, pristine WS_2_ devices usually exhibit relatively fast carrier recombination and limited persistent photoconductive behavior, which are unfavorable for the realization of pronounced synaptic memory and temporal plasticity.^19^^,^^20^ Therefore, an effective interfacial modulation strategy is needed to regulate carrier relaxation and charge retention in WS_2_, thereby enabling pronounced synaptic memory and temporal plasticity in optoelectronic synapses.

Molecular functionalization offers a practical means to tailor the interfacial electronic properties of 2D semiconductors. Organic dye molecules possess rich electronic states, strong optical absorption, and versatile interfacial interactions with layered materials.^21^^,^^22^^,^^23^ In particular, Rhodamine 6G (R6G) has been widely employed in hybrid optoelectronic systems because of its favorable energy-level alignment and its capability to participate in interfacial charge transfer.^24^^,^^25^^,^^26^ Previous studies have shown that dye/2D-material hybrid structures can enhance photoresponse through molecular sensitization, interfacial carrier separation, and trap-assisted carrier modulation.^27^^,^^28^^,^^29^ Nevertheless, for WS_2_/R6G hybrid systems, the relationship between interfacial exciton/carrier dynamics and synaptic photoresponse remains insufficiently clarified, especially from the perspective of ultrafast spectroscopy.

In this work, we investigate a WS_2_/R6G hybrid heterojunction photodevice and demonstrate that molecular modification can effectively induce synaptic photoresponse under light stimulation. Compared with pristine WS_2_, the hybrid device exhibits enhanced photoinduced current, more pronounced excitatory postsynaptic current (EPSC) behavior, and improved plasticity-related characteristics under pulsed illumination. By combining structural characterization, steady-state optical spectroscopy, time-resolved carrier-dynamics analysis, and electrical measurements, we show that the introduction of R6G modifies the interfacial electronic coupling and carrier relaxation pathways in WS_2_. The resulting charge transfer contributes to prolonged photoresponse and history-dependent conductance modulation. This study not only provides insight into the physical mechanism underlying synaptic behavior in molecule/TMD hybrid systems but also offers a practical strategy for designing low-dimensional optoelectronic synapses and multifunctional photodetectors.

## Results

### Synapse-like photoresponse of the WS_2_/R6G hybrid device

**Figure 1:**
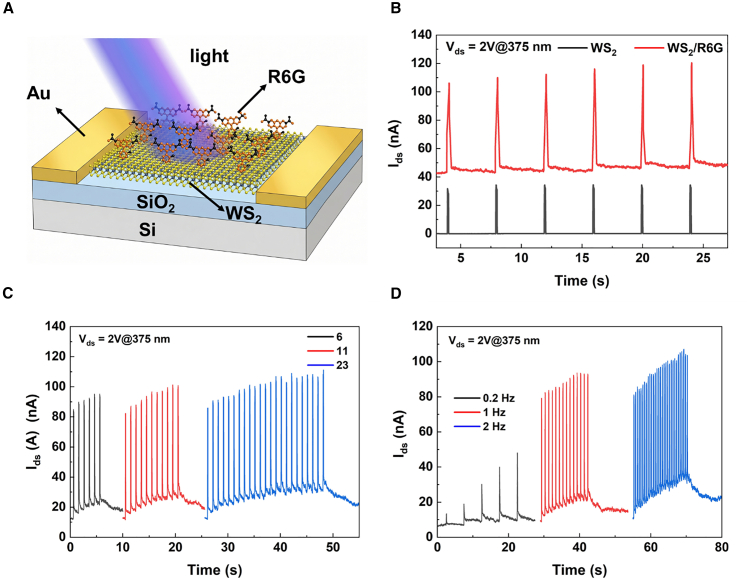
Synapse-like photoresponse of the WS_2_/R6G hybrid device (A) Schematic illustration of the WS_2_/R6G hybrid heterostructure-based device. (B) Time-dependent photocurrent responses of pristine WS_2_ and WS_2_/R6G devices under 375 nm pulsed light illumination. The WS_2_/R6G curve is vertically shifted upward by 34 nA to facilitate visual comparison. (C) Pulse-number-dependent EPSC responses of the WS_2_/R6G device. (D) Frequency-dependent photoresponse of the WS_2_/R6G device under repetitive optical stimulation.

To evaluate the optoelectronic synaptic functionality of the hybrid structure, the photoresponse behavior of the WS_2_/R6G device was investigated under 375 nm optical stimulation with a laser power density of 78 mW cm^−2^ and a pulse width of 200 ms. As illustrated in Figure 1A, an R6G molecular layer was introduced onto the surface of the WS_2_ channel bridging the Au source and drain electrodes on a SiO_2_/Si substrate. Figure 1B presents the pristine WS_2_ device exhibits only a weak photocurrent response with rapid decay under pulsed light illumination. In contrast, the WS_2_/R6G hybrid device shows a markedly enhanced current response together with a pronounced residual current after the light is turned off. The simultaneous appearance of enhanced photocurrent and delayed decay demonstrates that, after assembly with R6G, the device not only shows improved photocarrier generation and separation efficiency under illumination but also exhibits an obvious photomemory effect associated with prolonged carrier relaxation, analogous to the EPSC in biological synapses.^30^^,^^31^^,^^32^

The pulse-number-dependent EPSC response shown in Figure 1C further confirms the synapse-like characteristics of the device. With increasing pulse number, both the peak current and the accumulated residual current gradually increase, indicating that photogenerated carriers cannot fully recombine or de-trap during repeated optical stimulation. As a result, the postsynaptic current can be continuously modulated by the history of optical input, which is an important feature of neuromorphic optoelectronic devices.

Figure 1D presents the frequency-dependent current response of the WS_2_/R6G device under repetitive optical pulse stimulation. At a low stimulation frequency, the photocurrent nearly recovers to its initial level before the arrival of the subsequent pulse. However, with increasing pulse frequency, the current gradually accumulates and remains at a higher response level. This temporal summation behavior suggests that photogenerated carriers cannot fully recombine or de-trap within the pulse interval, leading to progressive current buildup. Such frequency-dependent plasticity is a hallmark of artificial synaptic devices, demonstrating that the WS_2_/R6G hybrid structure can effectively emulate biological synaptic functions under optical stimulation. In addition, paired-pulse facilitation was observed under two consecutive 375 nm light pulses, and the PPF index gradually decreased with increasing pulse interval, further supporting the short-term synaptic plasticity of the WS_2_/R6G device (Figure S1).

### Optical characterization of the WS_2_/R6G hybrid structure

**Figure 2:**
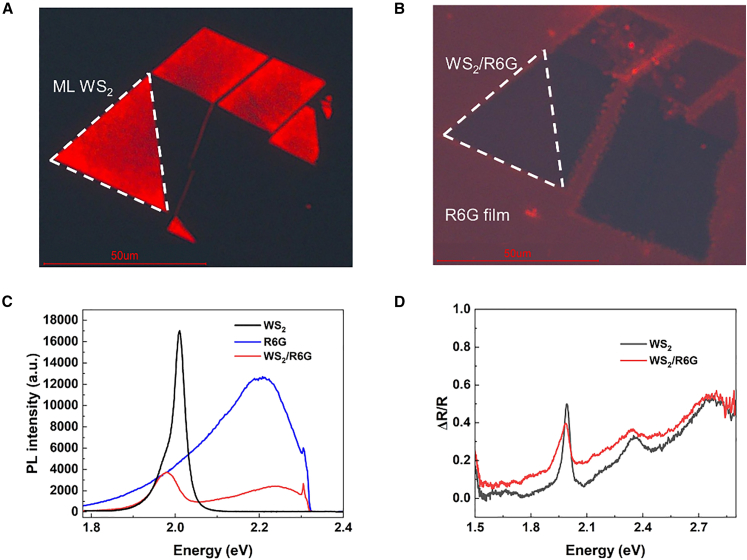
Optical characterization of the WS_2_/R6G hybrid structure (A and B) Photoluminescence image of monolayer WS_2_ (A) and WS_2_/R6G heterostructure (B). (C) Photoluminescence spectra of pristine WS_2_, R6G, and the WS_2_/R6G heterostructure. (D) Differential reflectance spectra of pristine WS_2_ and the WS_2_/R6G hybrid structure.

After establishing the synapse-like photoresponse of the WS_2_/R6G device, the structural characteristics and interfacial coupling of the hybrid system were further examined by fluorescence imaging and optical spectroscopy. As shown in Figure 2A, monolayer WS_2_ exhibits a well-defined triangular morphology. Raman characterization further confirms the characteristic vibrational features of the pristine WS_2_ flake (Figure S2). After R6G modification, the WS_2_ flake remains clearly identifiable and is surrounded by an additional molecular film, confirming the formation of the WS_2_/R6G hybrid structure, as shown in Figure 2B. The photoluminescence spectra in Figure 2C show pronounced quenching in the hybrid relative to pristine WS_2_ and pure R6G, indicating the introduction of additional nonradiative relaxation pathways after hybridization.^33^^,^^34^ Meanwhile, the WS_2_-related emission feature exhibits an apparent redshift, suggesting interfacial charge transfer and charge redistribution.^35^^,^^36^^,^^37^ As further shown in Figure 2D, the differential reflectance signal of the hybrid is enhanced, and the A-exciton feature near 2.0 eV becomes broadened after R6G modification, indicating modified excitonic properties and strengthened interfacial coupling. Collectively, these results confirm the existence of interfacial charge transfer in the WS_2_/R6G heterostructure, accompanied by charge redistribution.^38^^,^^39^

### Ultrafast carrier dynamics and interfacial charge transfer

**Figure 3:**
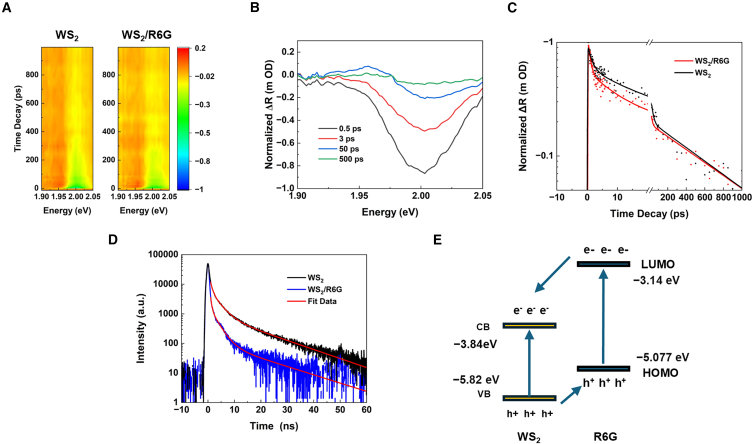
Carrier dynamics and interfacial charge transfer in the WS_2_/R6G hybrid structure (A) Normalized transient reflectance maps of pristine WS_2_ and WS_2_/R6G. (B) TR spectra of WS_2_/R6G at different delay times. (C) TR kinetic traces with tri-exponential fitting. (D) TRPL decay curves with fitting results. (E) Schematic energy-level alignment and interfacial charge-transfer process in WS_2_/R6G.

To clarify the carrier-dynamics mechanism underlying the enhanced photoresponse and synapse-like behavior of the WS_2_/R6G hybrid device, transient reflectance (TR) and time-resolved photoluminescence (TRPL) measurements were performed on pristine monolayer WS_2_ and the WS_2_/R6G hybrid structure. In the TR measurements, a 590 nm pump was used to largely avoid direct excitation of R6G, thereby mainly probing the evolution of the exciton population in WS_2_. This interpretation is further supported by the ultraviolet-visible (UV-vis) absorption results of the R6G solution and drop-cast R6G film (Figure S3). As shown in Figure 3A, both pristine WS_2_ and WS_2_/R6G exhibit a pronounced negative signal at approximately 2.0 eV, which can be assigned to the ground-state bleaching (GSB) of the WS_2_ A exciton. After R6G modification, the overall TR spectral profile remains almost unchanged, while the bleach recovery becomes significantly faster, indicating accelerated depletion of photoexcited excitons in WS_2_. Figure 3B shows the TR spectra of WS_2_/R6G at different delay times, where the A-exciton bleaching signal gradually recovers with increasing delay time. Furthermore, the normalized kinetic traces in Figure 3C show that WS_2_/R6G recovers faster than pristine WS_2_. Tri-exponential fitting yields decay time constants of τ_1_ = 1.372 ps, τ_2_ = 18.83 ps, and τ_3_ = 729.7 ps for pristine WS_2_, whereas τ_1_ and τ_2_ are shortened to 0.738 ps and 11.66 ps in WS_2_/R6G, with τ_3_ remaining nearly unchanged at 760.8 ps. These results indicate that R6G modification mainly accelerates the early-stage exciton depletion process and provides an additional interfacial relaxation pathway for photoexcited excitons in WS_2_.

The TRPL results further confirm that R6G modification modulates the exciton relaxation process in WS_2_. As shown in Figure 3D, the PL decay curves of pristine WS_2_ and WS_2_/R6G can both be well fitted using a tri-exponential model. The lifetime components of pristine WS_2_ are 0.632, 2.925, and 14.2 ns, respectively. After R6G modification, the fast and intermediate components are shortened to 0.399 and 2.534 ns, while the long-lived component is slightly prolonged to 15.494 ns. The pronounced shortening of the fast and intermediate lifetime components indicates enhanced interfacial coupling and accelerated hole transfer from photoexcited WS_2_ to R6G, which provides an additional nonradiative relaxation pathway and promotes early-stage exciton depletion. In contrast, the slightly prolonged long-lived component can be attributed to the slower recombination of residual electrons in WS_2_ after hole transfer to R6G. This spatial charge separation suppresses rapid electron-hole recombination and extends the carrier lifetime at the late relaxation stage. Combined with the energy-level alignment diagram shown in Figure 3E, the favorable matching between the valence band of WS_2_ and the HOMO level of R6G makes hole transfer from WS_2_ to R6G energetically feasible.^40^^,^^41^ Therefore, the accelerated TR recovery, shortened fast/intermediate TRPL components, and slightly prolonged long-lived component together support an interfacial hole-transfer-induced charge-separation mechanism in the WS_2_/R6G hybrid structure. This process modulates the exciton relaxation pathways in WS_2_ and provides a dynamical basis for the enhanced ultraviolet photoresponse and synapse-like photomemory behavior of the device.

### Enhanced UV photodetection performance

**Figure 4:**
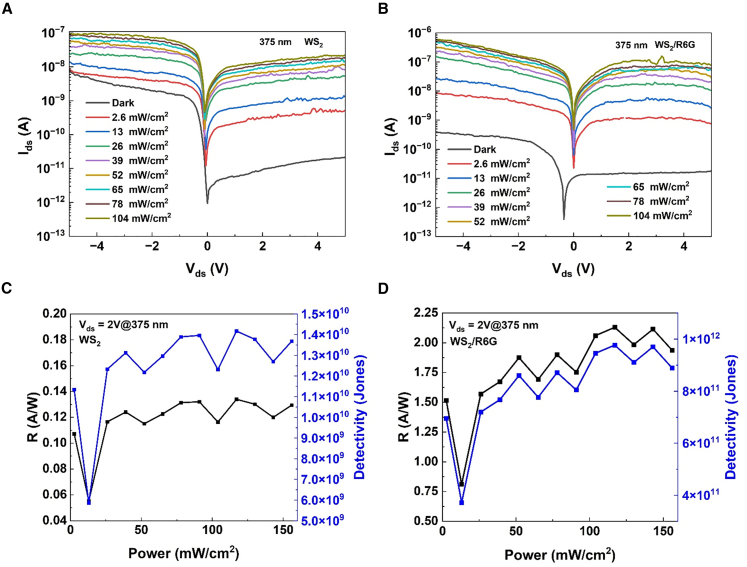
Photodetection performance of pristine WS_2_ and WS_2_/R6G devices under 375 nm illumination (A and B) Photodetection performance of pristine WS_2_ and WS_2_/R6G devices under 375 nm illumination. I_ds_-V_ds_ curves of pristine WS_2_ (A) and WS_2_/R6G (B) under different light power densities. (C and D) Responsivity and detectivity of pristine WS_2_ (C) and WS_2_/R6G (D) as functions of light power density at 2 V bias.

Figure 4 further evaluates the UV photodetection performance of pristine WS_2_ and WS_2_/R6G hybrid devices under 375 nm illumination. Figures 4A and 4B show the I-V characteristics measured in the dark and under different light power densities. For both devices, the drain current increases monotonically with increasing optical power, indicating their pronounced response to ultraviolet excitation. In addition, the power-dependent photoresponse was further fitted using a power-law relationship, as shown in (Figure S4). Compared with pristine WS_2_, the WS_2_/R6G hybrid exhibits a much higher current level over the entire bias range under identical illumination conditions. This enhancement is consistent with the shortened fast and intermediate exciton lifetimes observed in WS_2_/R6G, suggesting that R6G modification promotes exciton dissociation and interfacial carrier separation through an efficient hole-transfer process.

The enhanced photoresponse is further quantified by the responsivity (R) and specific detectivity (D∗) summarized in Figures 4C and 4D, respectively, measured at V_ds_ = 2V under 375 nm illumination. The pristine WS_2_ device shows relatively low responsivity and detectivity with only weak dependence on optical power. In contrast, the WS_2_/R6G hybrid shows substantially improved photodetection performance, with the maximum responsivity increasing from 0.133 to 2.13 A/W and the detectivity rising from 1.42 × 1010 to 9.77 × 1011 Jones after R6G modification.

Such enhancement can be attributed to the interfacial coupling between WS_2_ and R6G. After R6G modification, a type-II heterojunction-like band alignment favorable for charge separation is formed at the WS_2_/R6G interface. Under optical excitation, photogenerated electrons and holes are spatially separated by the interfacial energy-level offset and distributed in the WS_2_ layer and the R6G molecular layer, respectively, which reduces the probability of direct electron-hole recombination and increases the effective carrier population contributing to the photocurrent.

### Dynamic photoresponse and persistent photoconductive behavior

**Figure 5:**
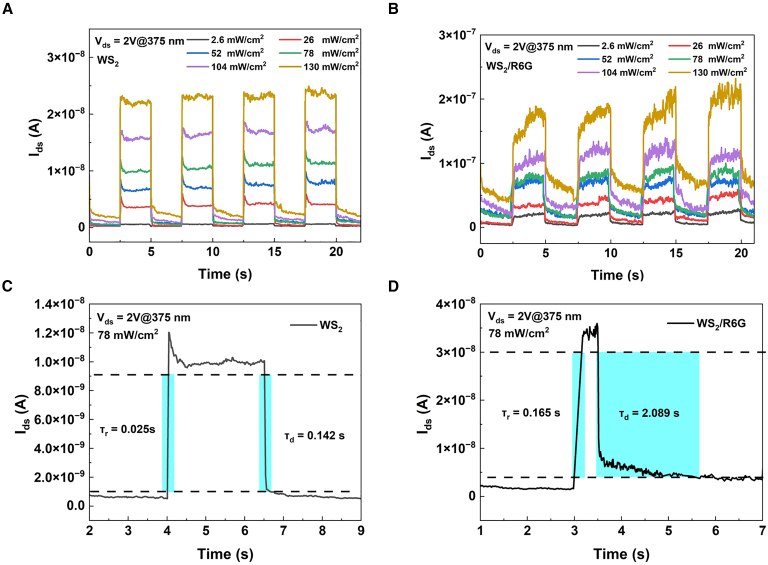
Dynamic photoresponse of pristine WS_2_ and WS_2_/R6G devices under 375 nm illumination (A) Time-dependent photoresponse of the pristine WS_2_ device under different light power densities. (B) Time-dependent photoresponse of the WS_2_/R6G hybrid device under different light power densities. (C) Single-pulse response of the pristine WS_2_ device at 78 mW/cm^2^. (D) Single-pulse response of the WS_2_/R6G hybrid device at 78 mW/cm^2^, showing prolonged rise and decay processes after R6G modification.

To further compare the dynamic photoresponse behaviors of pristine WS_2_ and WS_2_/R6G hybrid devices, the switching characteristics under different optical power densities were investigated, as shown in Figure 5. Figures 5A and 5B present the I-T curves of the WS_2_ and WS_2_/R6G devices, respectively, measured under 375 nm illumination at V_ds_ = 2 V. As the light power density increases from 2.6 to 130 mW/cm^2^, the photocurrent of both devices increases gradually, indicating that stronger illumination generates more photocarriers and thus enhances the photoresponse.

In comparison, the WS_2_/R6G hybrid device exhibits a significantly higher photocurrent and a much longer decay after the light is turned off. This behavior suggests that R6G modification strongly affects carrier transport and relaxation through interfacial coupling. The enhanced and delayed photoresponse indicates the existence of additional charge transfer processes in the hybrid structure, leading to persistent photoconductive behavior. This feature is consistent with the synaptic photoresponse discussed previously, indicating that the interfacial modification not only improves the response intensity but also endows the device with a more pronounced memory effect.

To quantitatively evaluate the response speed, the rise time and decay time under a single optical pulse were extracted. For the pristine WS_2_ device, the rise time T_r_ and decay time T_d_ are 0.025 and 0.142 s under 78 mW/cm^2^ illumination, respectively, as shown in Figure 5C. For the WS_2_/R6G hybrid device, representative transient analysis yields a rise time (T_r_) increases to 0.165 s and a decay time (T_d_) is significantly prolonged to 2.089 s, as shown in Figure 5D. The greatly extended decay process further confirms that the introduction of R6G promotes interfacial charge transfer, thereby leading to a stronger persistent photoresponse and more pronounced synaptic memory behavior.

## Discussion

In this work, a WS_2_/R6G hybrid device was fabricated to investigate the interfacial modulation of ultraviolet photoresponse and synaptic optoelectronic behavior. The hybrid structure exhibits enhanced photocurrent response, persistent current retention, and characteristic frequency- and intensity-dependent synaptic plasticity under 375 nm optical stimulation. Structural and spectroscopic analyses confirm the formation of the WS_2_/R6G interface and reveal strong interfacial electronic coupling between WS_2_ and R6G. In particular, the quenched photoluminescence, broadened excitonic resonance, accelerated TR recovery, and shortened time-resolved photoluminescence lifetimes consistently demonstrate that R6G modification induces efficient interfacial charge transfer in the WS_2_/R6G heterostructure, leading to rapid depletion of photoexcited WS_2_ excitons. This interfacial charge transfer promotes the formation of a long-lived charge-separated state. As a result, the hybrid device shows substantially improved ultraviolet photodetection performance, with the maximum responsivity increasing from 0.133 to 2.13 A/W and the detectivity increasing from 1.42 × 10^10^ to 9.77 × 10^11^ Jones. These results demonstrate that molecular functionalization provides an effective strategy for regulating interfacial charge transfer and carrier dynamics, thereby enabling multifunctional WS_2_-based optoelectronic synaptic devices.

### Limitations of the study

Although this study demonstrates that the WS_2_/R6G hybrid strategy can effectively enhance ultraviolet photoresponse and enable synapse-like optoelectronic behavior, several limitations should still be acknowledged. The present work mainly focuses on device performance under 375 nm optical stimulation and a limited range of bias conditions. Therefore, the photoresponse and synaptic plasticity of the hybrid device over broader spectral ranges, at lower optical power densities, and under different pulse-parameter conditions remain to be further investigated. In addition, the WS_2_ flakes used in this study were obtained by mechanical exfoliation, which is suitable for fundamental single-device demonstrations but remains limited in terms of large-area fabrication and array-level integration. Furthermore, the long-term operational stability, environmental robustness, and photochemical durability of R6G, as well as the cycling endurance of the WS_2_/R6G hybrid device, have not yet been systematically evaluated.

## Resource availability

### Lead contact

Requests for further information and resources should be directed to and will be fulfilled by the lead contact, Tengfei Yan (yantf@shu.edu.cn).

### Materials availability

This study did not generate new unique reagents.

### Data and code availability

• The data supporting the findings of this study are available within the manuscript and the supplemental information.
• This paper does not report original code.
• Any additional information required is available upon reasonable request to the lead contact.

## Acknowledgments

The authors acknowledge financial support by Shanghai Science and Technology Commission under grant no. 24142200200.

## Author contributions

W.X. and T.Y. designed the project. W.X. and Q.H. collected and analyzed the data. W.X., L.J., D.T., and T.Y. wrote the paper.

## Declaration of interests

The authors declare no conflict of interest.

## Declaration of generative AI and AI-assisted technologies in the writing process

During the preparation of this work, the authors used ChatGPT (OpenAI) to check spelling. The authors reviewed and edited the content and took full responsibility for the publication.

## STAR★Methods

### Key resources table

**Table:** 

REAGENT or RESOURCE	SOURCE	IDENTIFIER
**Chemicals, peptides, and recombinant proteins**		

Rhodamine 6G	MACKLIN	CAS:989-38-8
WS_2_	HQ graphene	CAS:12138-09-9

**Software and algorithms**		

OriginPro 2024	OriginLab Corporation	https://www.originlab.com/
Surface Xplorer	Ultrafast Systems	https://www.ultrafast.systems/products/spectrometers-accessories/surface-xplorer/

### Method details

#### Device fabrication

WS_2_ flakes were mechanically exfoliated from bulk crystals and picked up by a PDMS stamp. The selected flakes were then transferred onto a SiO_2_/Si substrate prepatterned with Au source and drain electrodes. The electrodes were fabricated by standard photolithography, thermal evaporation, and lift-off processes, thereby establishing electrical contact between the WS_2_ channel and the metal electrodes. To construct the hybrid device, an aqueous R6G solution with a concentration of 10^-6^ M was drop-cast onto the device surface. After 15 mins, the remaining solution was removed, yielding the WS_2_/R6G hybrid device.

#### Optoelectronic and synaptic measurements

The optical properties of the WS_2_ and WS_2_/R6G structures were characterized using a photoluminescence/reflectance spectroscopy setup. The electrical transport characteristics and synaptic photoresponse were measured with a Keithley 4200A semiconductor parameter analyzer. During the measurements, a bias voltage was applied across the source and drain electrodes while the device current was recorded simultaneously. A 375 nm laser was used as the optical stimulus, and the optical pulse sequence was controlled by a RIGOL DG822 arbitrary waveform generator.

#### Transient reflectance and time-resolved photoluminescence measurements

Transient reflectance (TR) measurements were performed under identical experimental conditions for both monolayer WS_2_ and WS_2_/R6G hybrid structures. A 590 nm femtosecond pulsed laser was used as the pump beam with an average power of 9 μW, while a supercontinuum white light served as the probe beam. The temporal window for the TR measurements was set to 1 ns. Time-resolved photoluminescence (TRPL) measurements were carried out using an Edinburgh Instruments FL1000 fluorescence spectroscopy system. A 375 nm pulsed laser was employed as the excitation source, and the emission signal was collected at 620 nm.

### Quantification and statistical analysis

No statistical methods were used to predetermine sample size, and no statistical hypothesis tests were performed in this study.

All statistical analyses and data processing were performed using OriginPro 2024 and Surface Xplorer software. Photoluminescence, differential reflectance, transient reflectance, time-resolved photoluminescence, and electrical measurement data were analyzed based on the original experimental datasets. Transient reflectance data were processed using Surface Xplorer, and the kinetic traces were extracted from the corresponding spectra. Time-resolved photoluminescence decay curves were fitted using multi-exponential decay functions.

### Additional resources

Our study has not generated or contributed to a new website/forum or has not been part of a clinical trial.
