# Supplementary material for: Interfacial coupling-modulated ultraviolet photoresponse in WS2/R6G hybrid structure with synapse-like characteristics
Source: iScience. 2026 Jun 30;29(7):116621. doi: 10.1016/j.isci.2026.116621 (PMC13378374; doi:10.1016/j.isci.2026.116621)
Supplement: Document S1. Figures S1–S4 [file mmc1.pdf]

iScience, Volume *29*

Supplemental information

Interfacial coupling-modulated ultraviolet photoresponse in WS_2/R6G hybrid structure with synapse-like characteristics

Wenyu Xu, Lin Jiang, Qinyong He, Daliao Tao, and Tengfei Yan

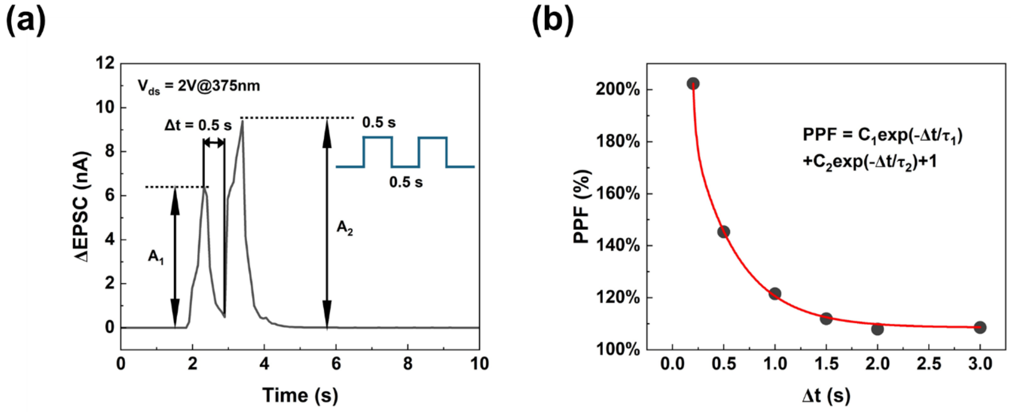

Figure S1. Synapse-like behaviour shown by the EPSC (excitatory postsynaptic current) and PPF (paired-pulse facilitation) photoresponse of the device. (a) EPSC produced by 375 nm light, pulse pair at a time interval of 0.5 s. (b) PPF variation as a function of Δt. PPF index is defined as (A_2/A_1) × 100%, in which A_2 and A_1 are the values of EPSC for the first pulse and the sequential pulse stimulation spikes, respectively. The PPF index decays as the pulse-to-pulse time interval (Δt) increases . The dependence of PPF on Δt is fitted by a bi-exponential decay function. The decay constants are 0.027 s and 0.448 s, respectively.

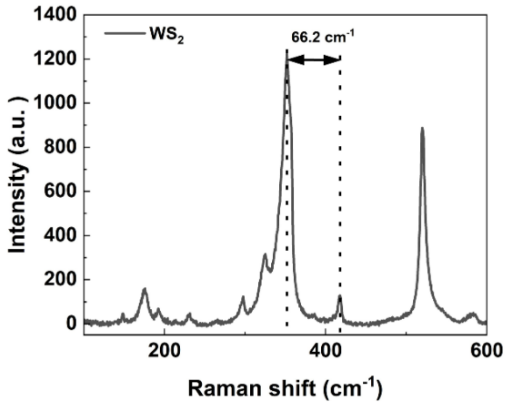

Figure S2. Raman characterization of pristine WS_2 sample. (a) Raman spectrum of monolayer WS_2 sample excited by a 532 nm laser.

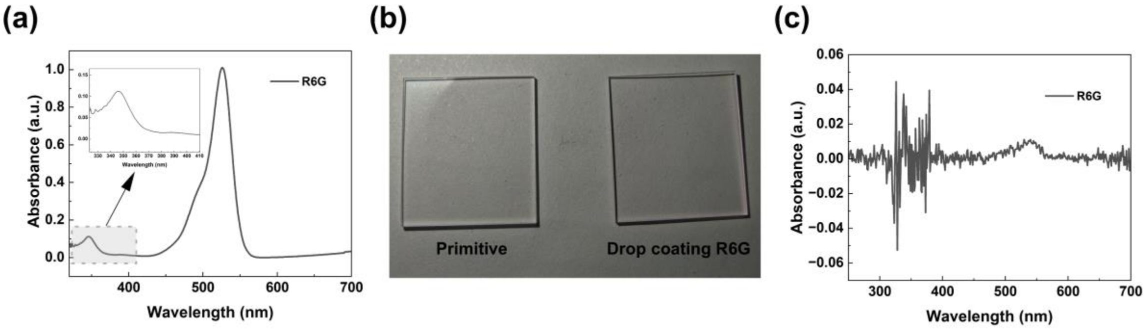

Figure S3. Absorption characterization of Rodamine 6G (R6G) samples. (a) UV-vis absorption spectrum of the R6G solution.(b) Photograph of the R6G film prepared on a quartz substrate using the same drop-casting procedure as that used for device fabrication.(c) UV–vis absorption spectrum of the drop-cast R6G film.

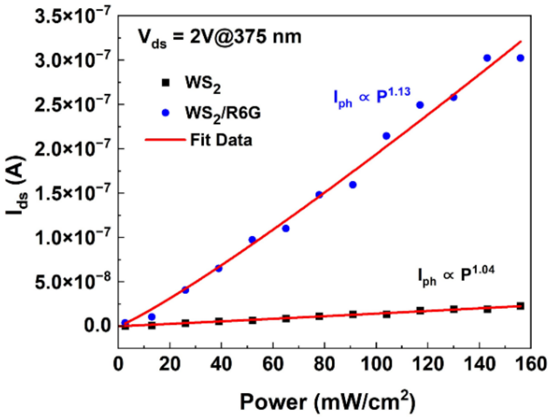

Figure S4. Photoresponse of devices at different excitation powers. (a) Power-dependent photocurrent of pristine WS_2 (black dots) and WS_2/R6G (blue dots) devices under 375 nm illumination, respectively. The solid lines are power law fits to the data.
